# Gelatin-grafted tubular asymmetric scaffolds promote ureteral regeneration *via* activation of the integrin/Erk signaling pathway

**DOI:** 10.3389/fbioe.2022.1092543

**Published:** 2023-01-05

**Authors:** Baiyang Song, Li Fang, Xufeng Mao, Xianwang Ye, Zejun Yan, Qi Ma, Zewen Shi, Yiwei Hu, Yabin Zhu, Yue Cheng

**Affiliations:** ^1^ School of Medicine, Ningbo University, Ningbo, China; ^2^ Department of Urology, Ningbo First Hospital, Ningbo, China; ^3^ Ningbo Clinical Research Center for Urological Disease, Ningbo, China; ^4^ Department of Radiology, Ningbo First Hospital, Ningbo, China

**Keywords:** ureter, tissue engineering, gelatin, urothelialization, MAPK/ERK pathway

## Abstract

**Introduction:** The repair of a diseased ureter is an urgent clinical issue that needs to be solved. A tissue-engineered scaffold for ureteral replacement is currently insufficient due to its incompetent bioactivity, especially in long-segment abnormalities. The primary reason is the failure of urothelialization on scaffolds.

**Methods:** In this work, we investigated the ability of gelatin-grafted tubular scaffold in ureteral repairment and its related biological mechanism. We designed various porous asymmetric poly (L-lactic acid) (PLLA)/poly (L-lactide-co-e-caprolactone) (PLCL) tubes with a thermally induced phase separation (TIPS) method *via* a change in the ratio of solvents (named PP). To regulate the phenotype of urothelial cells and ureteral reconstruction, gelatin was grafted onto the tubular scaffold using ammonolysis and glutaraldehyde crosslinking (named PP-gel). The *in vitro* and *in vivo* experiments were performed to test the biological function and the mechanism of the scaffolds.

**Results and Discussion:** The hydrophilicity of the scaffold significantly increased after gelatin grafting, which promoted the adhesion and proliferation of urothelial cells. Through subcutaneous implantation in rats, PP-gel scaffolds demonstrated good biocompatibility. The *in vivo* replacement showed that PP-gel could improve urothelium regeneration and maintain renal function after the ureter was replaced with an ∼4 cm-long PP-gel tube using New Zealand rabbits as the experimental animals. The related biologic mechanism of ureteral reconstruction was detected in detail. The gelatin-grafted scaffold upgraded the integrin α6/β4 on the urothelial cell membrane, which phosphorylates the focal adhesion kinase (FAK) and enhances urothelialization *via* the MAPK/Erk signaling pathway.

**Conclusion:** All these results confirmed that the PP46-gel scaffold is a promising candidate for the constitution of an engineered ureter and to repair long-segment ureteral defects.

## 1 Introduction

Iatrogenic accidents are the most common cause of ureteral trauma and are most frequent during gynecological and ureteroscopic surgeries (Zhao et al., 2019; Kapetanos et al., 2022). Urologists face a tough challenge in addressing the issue of long-segment ureteral abnormalities. In the clinic, an end-to-end suture is usually employed to repair short ureteral defects. However, long ureteral defects require the transplantation of other tissues like the small intestine and blood vessels. As a result, various problems, such as infection, anastomotic stenosis, chronic renal failure, and a shortage of donor tissue, are associated with these techniques due to the different natures of donor organizations (de Jonge et al., 2014; Zamani et al., 2020; Kapetanos et al., 2022).

Great attention has been focused on ureteral tissue engineering as a potential alternative of autologous grafts. Many materials, like acellular matrices, natural polymers, and synthetic polymers, have been studied and developed (Liao et al., 2013; Meng et al., 2015; de Jonge et al., 2018; Zhao et al., 2019; Gundogdu et al., 2021). However, ureteral repair has not been optimized because of the frequent formation of the fistula or/and stenosis caused by the insufficient bio-features of materials.

Histologically, the ureteral tissue consists of the urothelial layer, lamina propria, and smooth muscle surrounded by fibrous tissue and trophoblastic vessels (Koch et al., 2015; Ramuta and Kreft, 2018; Zamani et al., 2020). Urothelialization is essential for ureteral substitutes because the urothelium is specific to urinary tract tissue and serves as a barrier to protect the underlying tissue from toxic components in the urine. The formation of tight junctions between urothelial cells and asymmetric unit membranes (AMU) are two crucial issues. The fibrosis, shrinkage, and stenosis occur soon after the replacement if urothelialization is insufficient, and the tight junction between urothelial cells is poor (Dalghi et al., 2020; Wang et al., 2020).

AMU, which is made up of uroplakin proteins (UPs), is a sign of urothelial maturation. The scaffold that is urothelialized by AUM will function as the ureter to maintain metabolic homeostasis such as inhibiting the permeability of uric acid, NH3, and other small electrolytes from urine (Saksena et al., 2016; Dalghi et al., 2020). However, tight junctions among uroepithelial cells act as barriers and prevent harmful substances and microorganisms from infiltration into deeper tissues (Dalghi et al., 2020). Therefore, both the AUM and junctions between cells play essential roles in transporting urine and protecting the body against harmful substances and microorganisms. This is why the engineered scaffolds as substitutes support the phenotype of cells and play bio-functions (Zhang et al., 2017; Wang et al., 2022).

It is still unclear how to guide the functionalization of urothelial cells on scaffolds and the related biological mechanisms are still unknown. For example, seeding cells, like autologous urothelial cells, bone marrow mesenchymal stem cells, or adipose-derived stem cells, on scaffolds before transplanting is an effective way to form a mature urothelium (El-Hakim et al., 2005; Zhao et al., 2012; Liao et al., 2013; Meng et al., 2015; Zhao et al., 2016). Zhao et al. (2019) reported that seeding urinary-derived stem cells onto an acellular vessel could significantly promote urothelium formation in a long-segment ureteral defect with New Zealand rabbits as the model animals. However, the complexity, expensive cost, lengthy time involved in the procedure, and unsure bio-safety in the human body hinder the scaffold from being used in the clinic.

Traditional synthetic polymers, like poly (L-lactic acid) (PLLA) and poly (L-lactide-co-e-caprolactone) (PLCL), are biodegradable and have been approved by the U.S. Food and Drug Administration (FDA) for use in humans (Tyler et al., 2016; Dethe et al., 2022). However, a lack of a cell adhesion domain in their molecules makes urothelialized impossible after they are implanted into the ureter *in situ* (Saksena et al., 2016; Xie et al., 2021). The surface grafting of biomacromolecules onto polymeric scaffolds is a useful method in promoting cell adhesion and advancing surface functionalization. In our previous work, we set up a technology using diamine aminolysis and glutaraldehyde crosslinking and grafted proteins onto polyesters like PLLA, PCL, and their copolymers. Quantitative analyses and bio-functions after protein grafting have been investigated in detail (Zhu et al., 2002; Zhu et al., 2004; Zhu and Chan-Park, 2007; Gong et al., 2013; Hou et al., 2016).

Gelatin is a partial hydrolysate of collagen and contains arginine–glycine–aspartate (RGD) peptide sequences (Liu et al., 2020). RGD peptide is able to modulate cells to adhere onto scaffolds and proliferate because it can be sensed by integrins on the cell membrane. Integrins are transmembrane heterodimers composed of *a* and *ß* subunits and play a bridge role in the signal transduction of focal adhesion between the cell membrane and extracellular matrix. Focal adhesion kinase (FAK) is a key intracellular signaling molecule that receives signals from external integrins and modulates downstream signal molecules involved in cell proliferation, differentiation, and migration (Dalton et al., 2020; Kim et al., 2020). Its activation is regulated by Tyr phosphorylation and occurs in response to the stimulation of integrins as well as the RTK and G protein-coupled receptor. FAK autophosphorylation occurs at Tyr 397, which activates the Src family kinase to mediate FAK’s actions such as FAK-mediated Ras signal transduction (Schlaepfer et al., 1994; Schlaepfer and Hunter, 1996; Dalton et al., 2020; Kim et al., 2020). Therefore, gelatin was grafted onto polymer to activate the scaffold biologic response.

**FIGURE 1:**
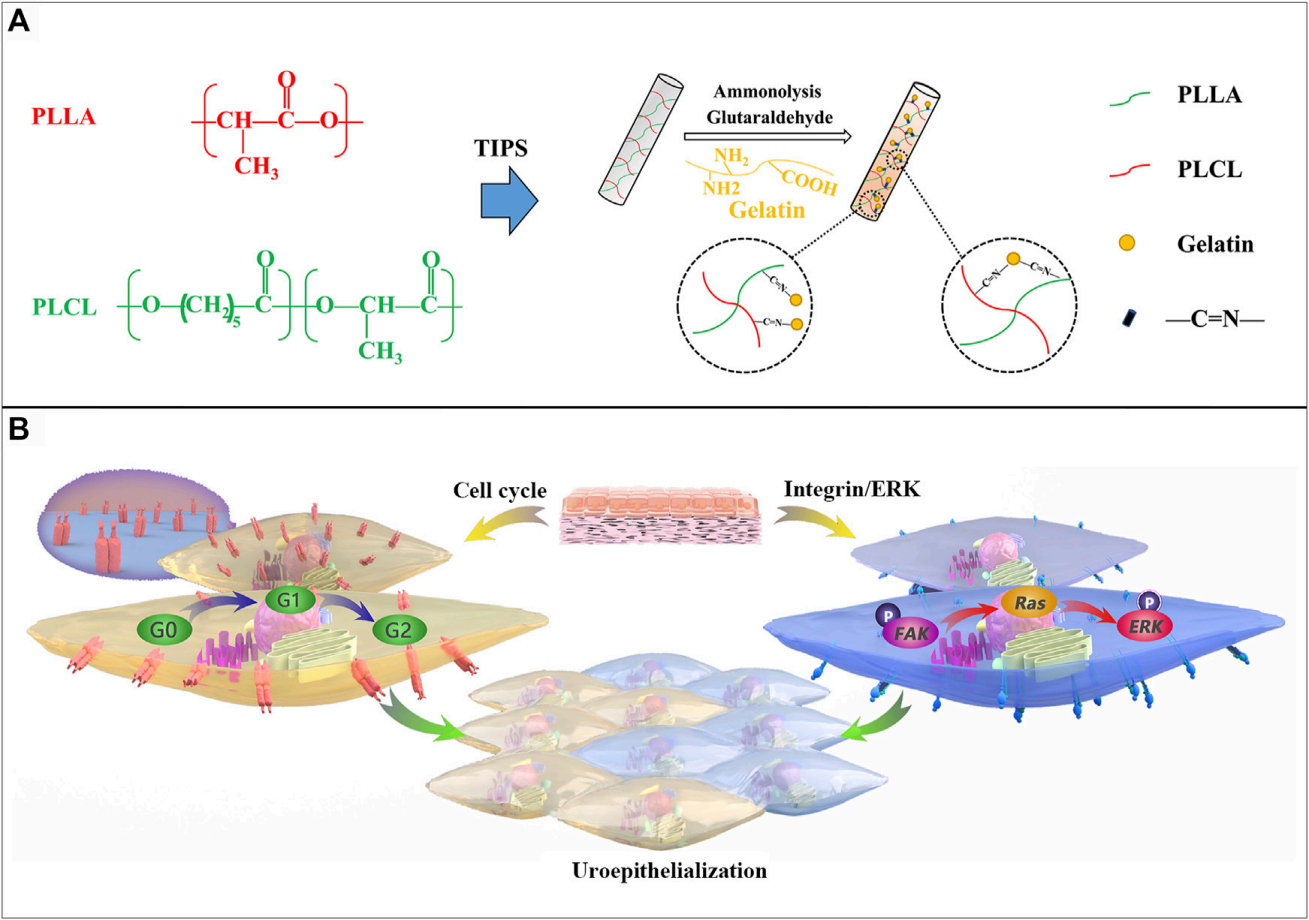
Procedure of PP-gel scaffold fabrication and the biomechanism of urothelialization. **(A)** Schematic illustration of the preparation of PLLA/PLCL scaffolds with grafted gelatin. **(B)** Biological mechanism of the PP-gel scaffold promoting urothelialization.

In this work, porous tubular scaffolds were fabricated with PLLA/PLCL mixtures in various material ratios and using a thermally induced phase separation (TIPS) method. It is important to note that the proportion of solvents affects the scaffolds’ microstructure. Gelatin was covalently bonded onto the scaffold using diamine aminolysis and glutaraldehyde crosslinking technology. The properties of the scaffold were examined by the adhesion, morphology, and proliferation of urothelium. The biocompatibility was tested *in vivo* using Sprague-Dawley rats as animal models. The feasibility of the scaffold as a ureter substitute was investigated with New Zealand rabbits as *in situ* implantation animals. The mechanism of ureter regeneration was determined, and Figure 1 demonstrates the procedure of PP-gel scaffold fabrication and the biomechanism of urothelialization. All findings demonstrate that the tissue-engineered PLLA/PLCL scaffold grafted with gelatin is a promising candidate as a ureter replacement. It is also expected that this technology will be employed in other human small-diameter tubular tissues in the future.

## 2 Materials and methods

### 2.1 Materials

PLLA (M_W_, 20 kDa) was a gift from the Ningbo Institute of Materials Technology and Engineering. PLCL (LA:CL, 50:50, M_W_ 30 kDa) was purchased from Daigang Biomaterials Inc. (Jinan, China). 1,4-Dioxane, 1,6-hexamethylenediamine (HMD) and glutaraldehyde (GA) were purchased from Aladdin Reagent Co. (Shanghai, China). Gelatin was purchased from Sigma-Aldrich Inc. (St Louis, Mo, United States). Immortalized human uroepithelial cells (HUC) were purchased from Procell Life Science & Technology Co., Ltd. (Wuhan, China).

### 2.2 Preparation of the porous scaffold

PLLA and PLCL were dissolved in dioxane at 65 °C various weight ratios (1:9, 2:8, 3:7, 4:6, 5:5, 4:6, 3:7, 2:8, and 1:9) to obtain a homogeneous solution with a final concentration of 10 g/ml. Then, it was poured onto a home-made mold, which contains a stainless rod (diameter, 1.0 mm) in the center and an exterior polytetrafluoroethylene cylinder (inner diameter, 3.0 mm) followed by heating at 65 °C. The mold was immediately placed in −80 °C and kept for 24 h followed by lyophilization for 72 h in a freeze dryer (Labconco, United States). Then, porous tubular scaffolds with 1.0 mm thickness were obtained, which were named PP19-PP91 according to the weight ratios of PLLA and PLCL components.

### 2.3 Gelatin-grafting on scaffolds

The aminolysis and GA cross-linking approach developed by our group was employed to immobilize gelatin onto the PP scaffold (Zhu et al., 2002; Zhu et al., 2004; Zhu and Chan-Park, 2007; Gong et al., 2013; Hou et al., 2016). Briefly, the scaffold was soaked in 95% alcohol for 1 h to eliminate impurities and then dried in a vacuum oven. Afterwards, it was immersed in 1,6-hexanediamine/isopropanol solution (0.06 g/ml) for 10 min at 37 °C followed by washing with water to remove the free hexanediamine. This aminolyzed scaffold was submerged in a GA aqueous solution (1.0 wt%) at ambient temperature for 3 h followed by rinsing with a large amount of water to remove the unreacted GA. Finally, the scaffold was cultured in a gelatin/PBS solution (1.0 wt%) for 24 h at room temperature followed by washing with water to remove the ungrafted gelatin. A polymeric scaffold grafted with gelatin (PP-gel) was finally achieved, and the experimental procedure is schematically displayed in Supplementary Figure S2A.

### 2.4 Scaffold characterization

#### 2.4.1 Porosity

The porosity of the scaffold was measured through an ethanol replacement method as described in the literature (Hou et al., 2016). Ethanol can get into pores but it will not make the scaffold shrink or swell. The tubular scaffolds were cut to a specific size with a height of 1 cm. *M*_*0*_ denoted its original weight, and this disc was weighed as *M*_*1*_ after it was placed in ethanol for 12 h to permit the ethanol to fill all the pores. The porosity of the disc was calculated as *P=*(*M*_*1*_ *-M*_*0*_)/(ρ*V), where *ρ* represents the density of ethanol. Each sample was repeated in triplicate.

#### 2.4.2 Microstructure measurement

The morphology of the scaffolds was observed using a scanning electron microscope (SEM). To acquire a cross-sectional sample, the scaffold was quenched in liquid nitrogen and then sputter-coated with gold using an Auto Fine Coater (E-1010, Hitachi, Japan). The scaffold morphology was observed under SEM (TM-3000, Hitachi, Japan) at a 10 kV accelerating voltage. The pore diameter and wall thickness were measured using ImageJ software using at least 50 locations of each image.

#### 2.4.3 Mechanical test

A mechanical test was performed using a universal material tester (Instron 3366, United States) at a deformation rate of 10 mm/min. Samples were cut from the tubular scaffolds with a dimension of 10 mm × 50 mm, and the thickness was measured using a micrometer with a precision of 0.01 mm. For each scaffold type, three replicates were tested.

The suture strength was measured as well, and the scaffold was fixed in the lower grip of the instrument and the suture was inserted from its outside edge (2 mm). These two ends of the suture were attached to the upper grip and pulled at a rate of 1 mm/min. The suture strength was defined as the maximum force (N). The tests were conducted in triplicate (*n* = 3) and the values were averaged.

#### 2.4.4 Contact angle measurement

The hydrophilicity before and after gelatin grafting was measured on a contact angle instrument (DataPhysics OCA20, Germany). Before measurement, the samples were dried overnight at 37 °C under vacuum. The measurement was conducted by dropping deionized water on the surface of the porous scaffold for 10 s. For each sample, three independent performances were collected.

#### 2.4.5 Fourier transform infrared (FTIR) spectroscopy

Attenuated total reflection Fourier transform infrared spectroscopy (ATR–FTIR) was performed using a Nicolet-670 FTIR spectrometer (Thermo, United States). All samples were measured at wavelengths from 1000 to 4000 cm^−1^ with a resolution of 4 cm^−1^.

### 2.5 Cell culture

HUCs were cultured in F12K medium (Gibco, United States) containing 10% fetal bovine serum (FBS, Gibco), 100U/mL penicillin, and 100 g/ml streptomycin (Gibco) at 37 °C in a humidified environment with 5% CO_2_. The medium was renewed every two days and subcultured when cells were confluent around 80%. For the biocompatibility tests, HUCs were seeded on PP and PP-gel scaffolds. A cell counting kit-8 assay (CCK-8) was used to detect the cell metabolic activity (*n* = 5), which was directly correlated with the cell number. At the same time, the cell number was counted using a hemocytometer (Bio-Rad, United States). A confocal laser microscope (CLSM, SP8, Leica, Germany) and a scanning electron microscope (SEM) were used to observe the distribution of HUCs on scaffolds. The cells were stained with F-actin and the nuclei were stained with phalloidin/4, 6-diamidino-2-phenylindole (DAPI).

### 2.6 Cell cycle analysis

The cells cultured on the scaffolds were harvested into a centrifuge tube and centrifuged at 1000 rpm for 5 min after being digested with 0.25% trypsin, and the supernatant was removed. Then, 1 ml of DNA staining solution (MultiSciences, China) and 10 μL of permeabilization solution (Multisciences, China) were added into a cell tube, stirred for 5 min with pipettes, and incubated at room temperature for 30 min in the dark. The fluorescent signals of cells were measured with flow cytometry (BD Accuri C6, United States). For each sample, three independent performances were collected.

### 2.7 Detection of EGF

HUCs were seeded on each scaffold at a density of 1.0 × 10^4^ per well (*n* = 5). After culturing for 3 or 7 days, the culture medium was collected. The amount of EGF in the medium was quantified using an EGF ELISA kit (RayBiotech, Inc., United States) according to the instructions.

### 2.8 Detection of cytokeratin AE1/AE3

HUCs were seeded onto scaffolds and cultured for 7 days. Then, the cells were fixed in 4% paraformaldehyde for 15 min and rinsed three times with PBS for 5 min each. To decrease non-specific background staining, the cells were incubated in 1% BSA for 20 min. The samples were then incubated overnight at 4 °C in mouse pan-cytokeratin antibodies AE1/AE3 (dilution 1:100; Santa Cruz). Subsequently, the samples were rinsed in PBS and incubated in FITC-conjugated goat anti-mouse secondary antibody (dilution 1:100; Cell Signaling Technology (CST)) for 1 h at room temperature. Finally, DAPI was used to label the cell nuclei. The samples were observed under a confocal laser scanning microscope (CLSM) to capture the visual results.

### 2.9 RNA isolation and real-time PCR performance

Real-time PCR (RT-PCR) was performed to evaluate the mRNA level of integrin α6 (ITGA6), integrin β4 (ITGB4), zonula occludens-1 (ZO-1), uroplakin Ⅲ (UP3), K-Ras, N-Ras, and H-Ras with GAPDH as the reference when cells were cultured for 7 days. The RNA was collected using the RNA reagent TRIzol (Invitrogen/Life Technologies, Carlsbad, CA) and dissolved in nuclease-free water. cDNA was synthesized using a PrimerScript RT reagent kit (TaKaRa, Dalian, China) according to the manual. Transcription and amplification were performed using SYBR Premix Ex Taq™ reagent (TaKaRa, Dalian, China). The cycling conditions were 95 °C for 30 s and 40 cycles including 95 °C for 5 s and 60 °C for 30 s. Data were analyzed using a comparative cycle threshold method in a Roche LightCycler480 System. The tests were conducted in triplicate (*n* = 3) and the values were averaged. The PCR primer sequences are listed in Supplementary Table S1.

### 2.10 Western blotting (WB)

Western blotting analysis was performed to quantitatively evaluate the expression level of integrin α6 (dilution 1:1000, Proteintech), integrin β4 (dilution 1:1000, Proteintech), FAK (dilution 1:1000, CST), p-FAK (Tyr397) (dilution 1:1000, CST), Ras (dilution 1:1000, CST), Erk (dilution 1:1000, CST), and p-Erk (dilution 1:1000, CST) when cells were cultured for 7 days. The GAPDH expression was used to standardize the data. Primary antibodies were incubated overnight at 4 °C followed by incubation of HRP-conjugated goat-anti-mouse secondary antibody for 1 h at room temperature. Image Lab was used to quantify the results, which were displayed as the relative expression to GAPDH. For each sample, three independent performances were collected.

### 2.11 *In vivo* tests

#### 2.11.1 Subcutaneous implantation

A total of 12 male SD rats were applied for this experiment. They were randomly divided into the PP46 and PP46-gel groups. Tubular scaffolds were cut into certain sized discs followed by sanitizing with 75% ethanol for 12 h and washing with PBS. SD rats were anesthetized by isoflurane inhalation, the hair was shaved, and the skin was disinfected with iodophor. A subcutaneous pocket on the right side of each animal’s back was made using surgical scissors. Then, the scaffold was inserted into the pocket. To avoid bacterial infection, penicillin was intramuscularly injected once a day for three days. After 4 or 8 w, the animals were sacrificed and the scaffolds together with the surrounding tissue were collected. Then, the sample was immersed in a 4% formalin solution and fixed for 24 h. After dehydration by graded ethanol solutions, the scaffolds were embedded in paraffin and microtomed into sections (4 μm). H&E, Masson, immunofluorescence (IF) staining, and immunohistochemistry (IHC) were performed to detect the bio-features of scaffolds. Anti-CD68 antibody (dilution 1:500; Proteintech) was used to investigate the behaviors of macrophages around the scaffold.

#### 2.11.2 Ureteral reconstruction *in vivo*

A total of 18 adult New Zealand male rabbits were divided randomly into three groups: PP, PP-gel, and the autograft. After the rabbits were anesthetized with pentobarbital (30 mg/kg animal), the median abdomen of the rabbit was cut open to expose the left ureter along the paracolic sulcus. The ureter was defected around 4 cm in length followed by replacement with PP46, PP46-gel, or an autograft tube, and 8-0 Vicryl sutures were used to make anastomosis between the scaffolds and the ureter ends. A 3 Fr homemade ureteral stent was inserted into the ureter lumen to avoid anastomotic leakage. The procedure was performed under an operating microscope. After 2 w post-operation, the stent was removed from the bladder incision. All rabbits were raised for 8 or 12 w. At the test time, animals were sacrificed and the grafts were collected for histological studies. HE, Masson, and IHC staining were performed to verify the functionality of the grafts. Anti-AE1/AE3 antibody was used to detect urothelial cells. Computed tomography (CT) and CT urography (CTU) were used to analyze the function of the reconstructed ureters in each group. Venous serum was collected by centrifugation of the inferior vena cava (4500 g, 20 min). Blood urea nitrogen (BUN) and creatinine (Cr) in the serum were examined according to the manual introduction.

### 2.12 Statistical analysis

Data are shown as the mean and standard error (SD). Intergroup comparisons were expressed using the unpaired *t*-test between two groups and one-way ANOVA followed by the Tukey *post hoc* analysis for comparison of the aforementioned two groups. Data were analyzed by SPSS version 20.0, and statistical significance was represented as ns: *p* > 0.05; *p < 0.05; and **p < 0.01.

## 3 Results and discussion

### 3.1 Morphology and structure of the scaffolds

PLLA is a biocompatible polymer, which has been frequently used as a scaffold matrix for tissue engineering research. It is a crystalline polyester with a glass transition temperature (Tg) of ∼60 °C (Hou et al., 2015). Thus, it is a stiff and brittle material at 37 °C, which is the human body temperature. In contrast, PLCL has excellent elasticity and flexibility (Wang et al., 2015). Therefore, in this work, PLLA was mixed with PLCL in various ratios as stated in the Methods section to make an elastic and good mechanical strength matrix where the tubular scaffold was fabricated.

**FIGURE 2:**
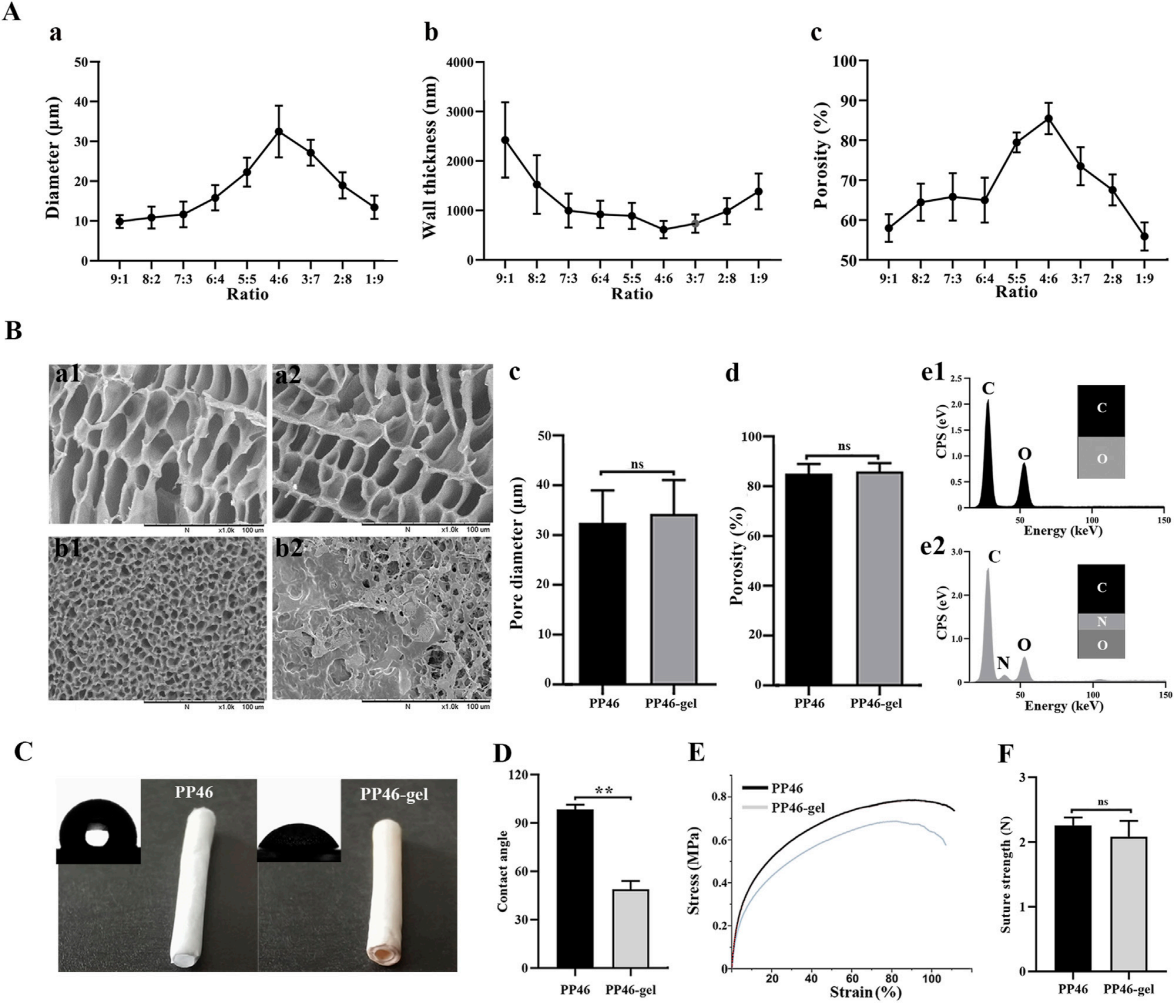
Characteristics of the PP46 and PP46-gel scaffold. **(A)** Pore diameter, wall thickness, and porosity as a function of the ratio of PLLA:PLCL. **(B)** Characteristics of the PP46 and PP46-gel. The morphology of scaffold PP46 and PP46-gel observed under SEM, cross section (a1-a2), and inner surface (b1-b2); **(C, D)** pore diameter and porosity of PP46-gel with scaffold PP46 as the reference (e1-e2). The EDS analysis of the inner surfaces of the PP46 and PP46-gel. **(C)** Contact angle alteration before and after gelatin grafting. **(D)** Quantitative analysis of the contact angles. **(E)** Stress–strain curves at a deformation rate of 10 mm/min. **(F)** Suture strength analysis. Data are presented as mean ± SD (**p < 0.01; ns: *p* > 0.05).

The merited percentage of PLCL and PLLA for the scaffold fabrication was investigated. All scaffolds, PP19-PP91, exhibited a porous structure (Supplementary Figure S1). In addition, the pore diameter on the inner surface of each scaffold was smaller than that on the cross section, which might be due to the unequal temperature drop rates during the TIPS process according to the reported literature (Zeinali et al., 2021). The small pores on the inner surface play a vital role in supporting urothelial cells for adhesion and growth. As shown in Supplementary Figure S1, the pore diameters of the inner surfaces of all scaffolds are similar, <10 μm, which is able to support the growth of the cells (Saksena et al., 2016; Lv et al., 2018). However, the bulk structure of the scaffolds, like pores and porosity, will influence the nutrient circulation, vessel formation, and regulation of inflammation (Feng et al., 2011; Wang et al., 2020; Zhang and King, 2022; Stahl et al., 2023). Among scaffolds PP19-PP91, PP46 exhibits the largest pores in the bulk (cross section, Supplementary Figure S1A4) of 32.48 ± 6.51 μm and the highest porosity at 85.48 ± 3.90% (Figures 2Aa–c). Therefore, material PP46 (4:6, PLLA:PLCL) was chosen as the matrix for the fabrication of the tubular scaffold for ureter tissue engineering research.

To promote the interaction between the scaffold and uroepithelial cells, gelatin was grafted onto the scaffold using the diamine aminolysis and glutaraldehyde crosslinking technology. From SEM observation, the morphologies of the cross-section of scaffolds were not altered much after gelatin-grafting (Figures 2Ba1, a2), like the pore diameter and porosity (Figures 2Bc–d), but the gelatin was found in the inner surface of the scaffold PP46-gel, which explained why elemental N was found on the surface of the PP gel scaffold through energy dispersive spectroscopy (EDS) (Figures 2Be1–e2). Furthermore, the chemistry of scaffold PP46 and PP46-gel was investigated with FTIR spectra (Supplementary Figure S2B). PP46-gel has two characteristic absorption peaks at 3396 cm^−1^ and 1622 cm^−1^, which are attributed to the hydroxyl and acylamino bonds from gelatin. In contrast, there are no similar peaks in the PP46 molecules. The absorption peak of the C=N bond, which is formed by the Schiff-base reaction, appears at 1680 cm^−1^ and is indicated by an arrow in the figure. These results verified that gelatin has been successfully grafted onto the PP46 scaffold. In addition, PP46-gel displayed good hydrophilicity; the contact angle decreased from 98.50 ± 2.93 ° of PP46 to 48.86 ± 5.20 ° and indicated the successful immobilization of gelatin onto the scaffold (Figures 2C, D). In addition, high hydrophilicity of the substratum promotes urothelial cell adhesion (Saksena et al., 2016).

The mechanical properties of the scaffolds were tested and compared after gelatin grafting with ungrafted PP46 as the control. Figure 2E represents the typical tensile stress-strain curves of PP46 and PP46-gel. The tensile strength and young’s modulus of PP46-gel decreased slightly: 0.78 ± 0.02 MPa for PP46 *vs.* 0.72 ± 0.03 MPa for PP46-gel (*p* < 0.05) and 8.69 ± 0.76 MPa for PP46 *vs.* 6.77 ± 0.82 MPa for PP46-gel (*p* < 0.05), which was attributed to the aminolysis process during gelatin grafting. The suture strength of the tube is a very important index. As shown in Figure 2F, the scaffolds display similar suture strengths with 2.26 ± 0.12 N for PP46 *vs.* 2.08 ± 0.25 N for PP46-gel (*p* > 0.05). The ester bond in the polymer molecule was aminolyzed to expose the free amino group (-NH_2_), through which gelatin can bond with GA *via* the Schiff base reaction. Nevertheless, the ultimate strain of the PP46-gel is similar to that of PP46 with 84.88 ± 3.22% for PP46 *vs.* 84.62 ± 3.09% for PP46-gel (*p* > 0.05). These results indicate that the mechanic strength of the scaffold was not greatly destroyed after being grafted by gelatin on the surface.

### 3.2 The behaviors of HUCs on scaffolds

**FIGURE 3:**
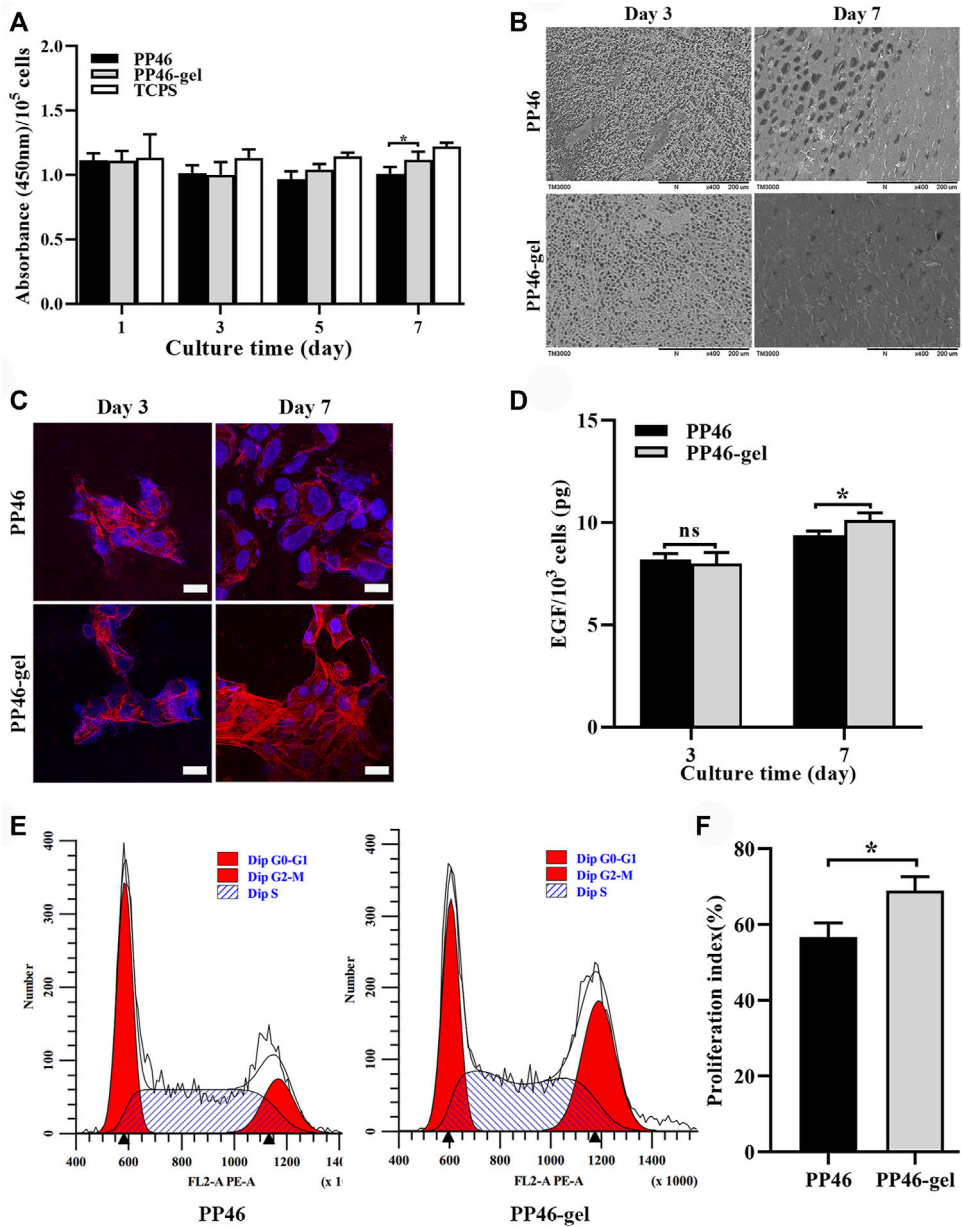
Proliferation of HUCs on the scaffold PP46 and PP46-gel. **(A)** Absorbance at a wavelength of 450 nm divided by the number of cells cultivated on the corresponding scaffolds. The cell number was measured with a hemocytometer. **(B)** Distribution of HUCs on the scaffolds observed under SEM. **(C)** Morphology of HUCs stained with F-actin (rhodamine-phalloidin, red) and nuclei (DAPI, blue). Scale bar, 20 μm. **(D)** Quantification of EGF secreted by cells on scaffolds PP46 or PP46-gel cultivated for 3 and 7 days, respectively. The value was divided by the number of cells cultivated on the corresponding scaffolds. **(E)** Cell cycle investigation tested using a flow cytometer. **(F)** Cell proliferation index (%) calculated from E. All data are presented as mean ± SD (*p < 0.05; ns: *p* > 0.05).

The proliferation of HUCs on PP46 and PP46-gel scaffolds was evaluated *via* tests of cells’ metabolic activity (CCK-8 method) with the cells on tissue culture polystyrene (TCPS) as the positive control. HUCs can grow and proliferate on both PP46 and PP46-gel during the culture time, which indicates that the scaffold material is not toxic for HUCs. To test the metabolic activity of cells growing on the different scaffolds, the absorbance at 450 nm was measured and converted by the cell number (measured using a hemocytometer) (Figure 3A). The results of CCK-8 analysis showed that at day 3, there was no significant difference in the absorbance on these two scaffolds. However, there were more living cells on the PP46-gel than on PP46 at day 5 and day 7 (*p* < 0.05) (Supplementary Figure S3). However, after the cell number deduction, the average metabolic activity of cells cultured on scaffolds were altered very little. There was no significant difference in the absorbance of each 10^5^ HUCs on these two scaffolds during the first 5 days. This indicated that the cells maintain the primary metabolic activity and are not deteriorated greatly after *in vitro* culture on the scaffolds. Nevertheless, cells cultured on PP46-gel displayed higher metabolic activity than those on PP46 at day 7 (*p* < 0.05), which indicated that the gelatin grafting is able to contribute to cell growth and proliferation.

From the SEM observation (Figure 3B), HUCs grew on both scaffold surfaces. Cells covered the pores partially on the scaffolds at day 3, but most pores were covered on day 7 with more cells on PP46-gel than on PP46. This result is consistent with that of the CCK8 assay (Figure 3A).

To observe the morphology of HUCs on the scaffolds, we conducted cytoskeleton immunofluorescence staining experiments. As shown in Figure 3C, cells on both scaffolds showed cytoskeleton but few numbers at day 3. At day 7, the number of cells on both scaffolds increased, but the cells on the PP46-gel showed normal morphology and expressed more actin bundles while the cells on PP46 were chaotic and spread out. Therefore, we speculate that the PP46-gel promoted HUCs proliferation and phenotypic expression.

EGF is an important cytokine that guides cells to transfer from the G0 to G1 phase and activate a new cell cycle (Jin et al., 2020). The EGF level secreted by cells on the PP46-gel (every 10^3^ HUCs in Figure 3D) is significantly higher than that on PP46 when they were cultured for 7 days; the value for the PP46-gel reached 9.39 ± 0.21 pg while it was 10.13 ± 0.36 pg for PP46 (Figure 3D), though there is little difference at day 3.

Subsequently, the distribution of the urothelial cell cycle was measured by flow cytometry. The results displayed that the cells starting at the G2/M stage on the PP46-gel are much more than those on PP46, while cells at the G0/G1 stage are similar at both scaffolds (left buns, Figure3E). The cell proliferation index calculated from [% = (S + G2/M)/(G0/G1+S + G2/M) × 100%] increased from 56.67 ± 3.75% for cells on PP46 to 68.99 ± 3.66% on the PP46-gel (Figure 3F), which indicated more cells on the PP46-gel than PP46 were growing and under mitosis. These results demonstrate that the gelatin-grafted PLLA/PLCL scaffold is able to promote HUCs growth and EGF secretion, which mediates cells undergo life cycles.

### 3.3 *In vivo* tests

#### 3.3.1 Subcutaneous implantation

**FIGURE 4:**
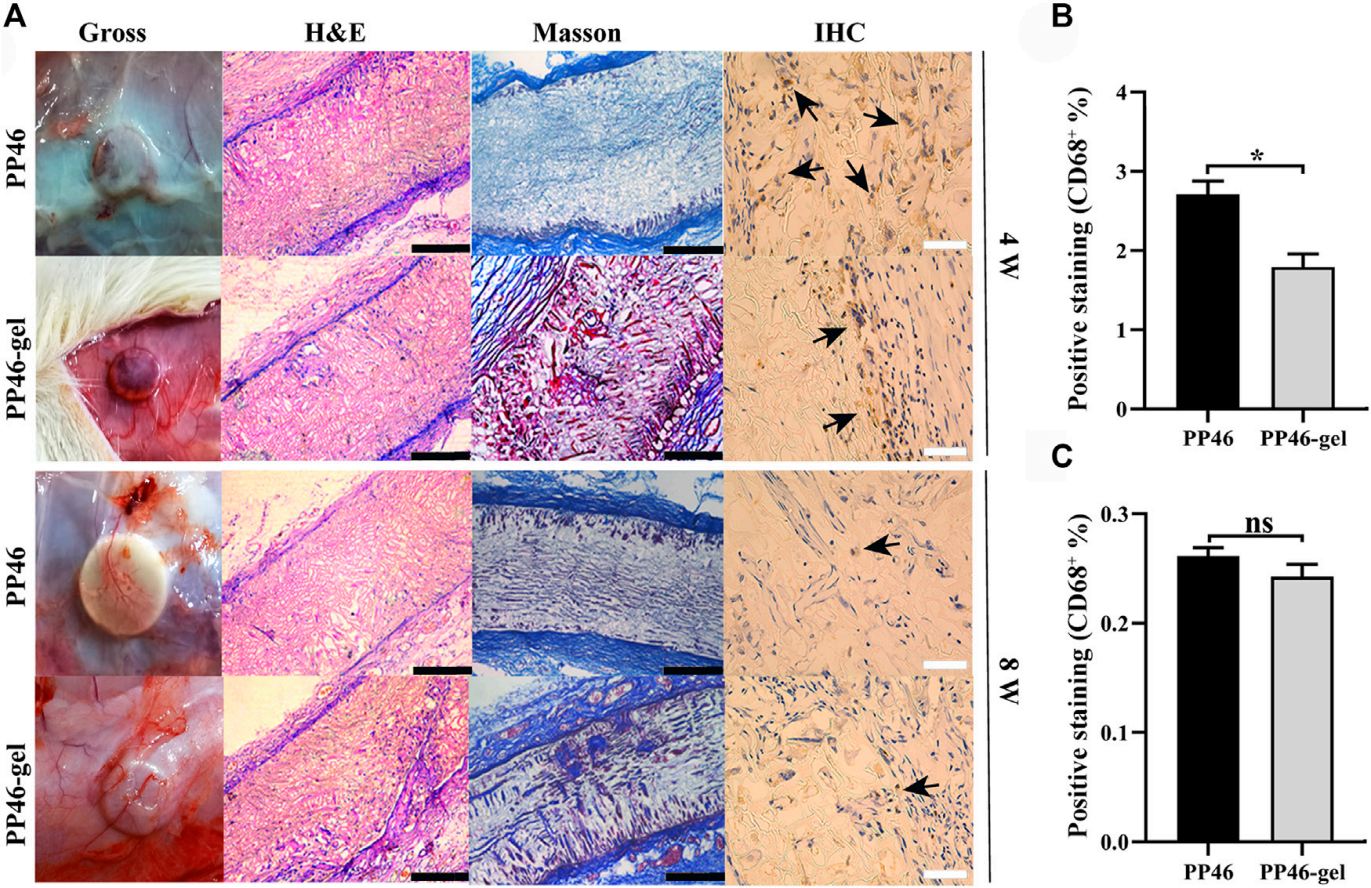
Histological analysis. **(A)** Scaffolds were implanted subcutaneously in SD rats for 4 and 8 weeks. Black arrows indicate CD68^+^ cells. **(B, C)** Quantitative analysis of the CD68^+^ ratio from IHC analysis. Scale bar, 400 μm (black) or 100 μm (white). Data are presented as mean ± SD (ns: *p* > 0.05; *p < 0.05).

PP46 and PP46-gel scaffolds were subcutaneously implanted in SD rats for 4 w and 8 w, respectively. The results of eye-sight observation showed that more blood vessels crossed over the PP46-gel scaffold than PP46 at both 4 w and 8 w. An adequate blood supplement is believed to be favorable for ureteral regeneration (Figure 4A, Gross). Correspondingly, more cells were integrated into the PP46-gel from the scaffold surface and more collagen was secreted over time from HE and Masson staining (Figure 4A, HE & Masson). Particularly, more cells and tissues proliferated inside the PP46-gel scaffold at 8 w, which shows that the PP46-gel recruited more cells and more tissues formed in the scaffold bulk compared with scaffold PP46.

CD68 is a specific marker of macrophages. The ratio of CD68^+^ reflects the inflammatory response of scaffolds (Al-Maawi et al., 2022). In our case, the inflammatory reaction mainly occurred at 4 w on both scaffold surfaces, and a higher ratio of CD68^+^ occurred at 4 w than at 8 w (Figures 4B, C). At 8 w, the inflammation around both scaffolds significantly decreased, which indicated that both PP46 and PP46-gel are biocompatible (Figure 4A, IHC). However, a lower ratio of CD68^+^ in the PP46-gel scaffold than that in PP46 at 4 w was observed in the semi-quantitative analysis (Figure 4B), which indicated that the period of the inflammatory stage was shortened after gelatin grafting. At 8 w, the ratio of CD68^+^ in the scaffolds significantly decreased, and there was no significant difference in the proportion of the positive area between them (Figure 4C). The results of immunofluorescent staining with anti-CD68 as the primary antibody displayed the same tendency with IHC (Supplementary Figure S4). The regression of inflammation is an inevitable requirement for tissue regeneration (Wynn and Vannella, 2016; Vannella and Wynn, 2017). Therefore, we conclude that the gelatin-grated scaffold alleviates the inflammatory response and shifts quickly into the proliferation stage, which is beneficial for the tissue/organ to repair or regenerate.

#### 3.3.2 Ureteral reconstruction *in vivo*

**FIGURE 5:**
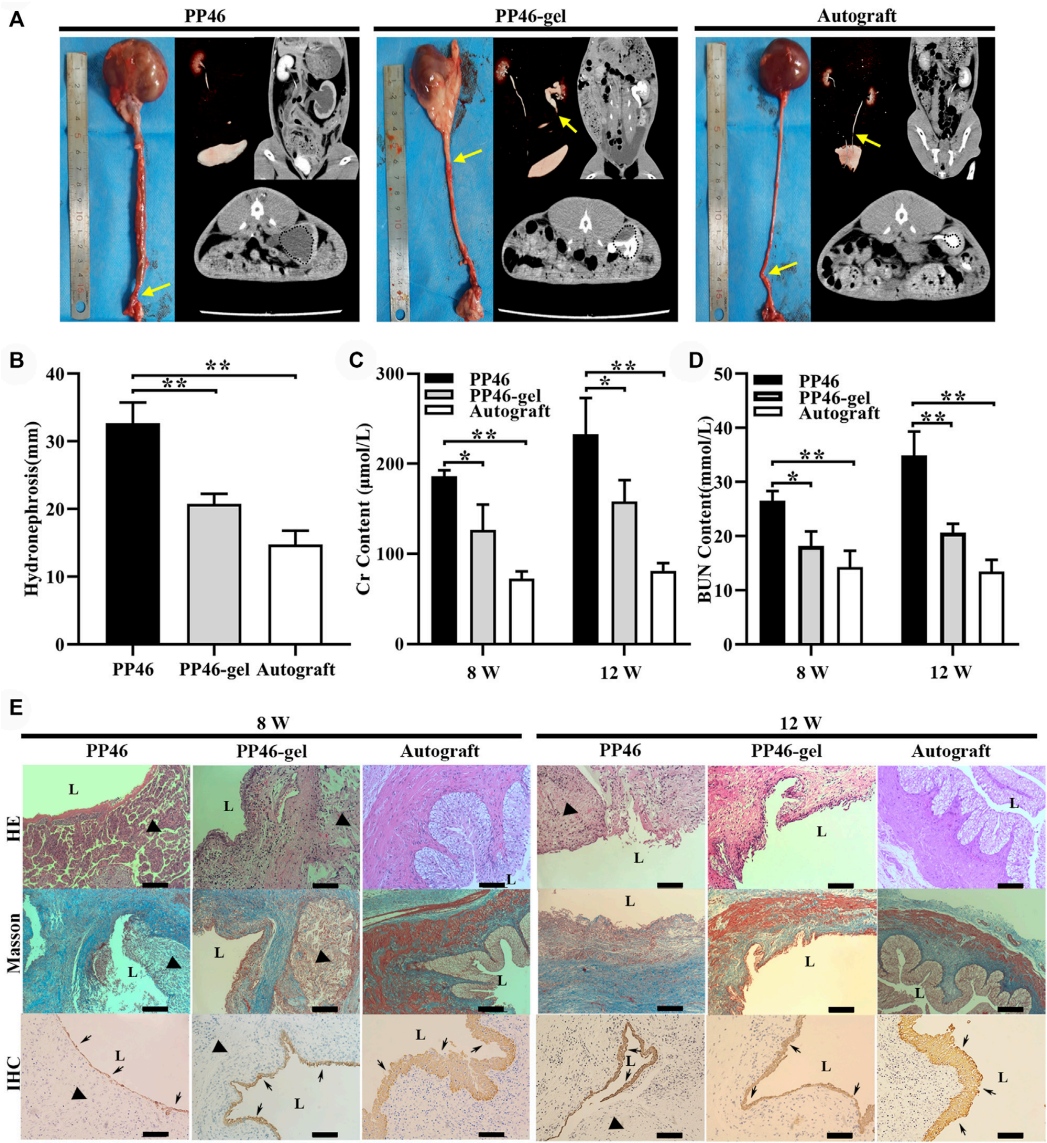
Results of the *in vivo* tests. **(A)** Gross and tomography of PP46 and PP46-gel with the autograft as the positive control. Yellow arrows denote ureteral obstruction. Dotted circles are hydronephrosis images in CT. **(B)** Degree of hydronephrosis evaluated from CT cross-sectional scanning postoperatively at 12 weeks. **(C, D)** Assessments of the Cr and BUN content after material implantation. **(E)** Histological analysis. L, lumen. The triangles indicate the location where scaffolds were implanted. Black arrows indicate AE1/AE3+ cells. Scale bar, 100 μm. Data are presented as mean ± SD (*p < 0.05; **p < 0.01).

Tubular scaffold PP46 and PP46-gel were grafted *in situ* with the autografting ureter as the positive control. After 12 w post-operation, a slight anastomotic stricture was observed at the surgery site in the autograft animals from the CT/CTU inspection (Figure 5A, yellow arrows); the values of Cr and BUN are the smallest among all three grafts (Figures 5C, D). This indicated that the renal function minimally deteriorated, and the upper urinary tract is minimally obstructed. For the PP46 and PP46-gel scaffolds, strictures existed at the surgery sites, but PP46-gel was only partially obstructed *vs.* completely obstructed PP46 (Figure 5A, arrows). In the animals whose ureter was substituted by the PP46 tube, the ureter and renal pelvis greatly dilated with the occurrence of hydronephrosis (Figure 5A, dotted circle), which indicates that the renal function was seriously damaged. While the ureter and renal pelvis in the PP46-gel group exhibited mild expansion and less serious hydronephrosis occurred (*p* < 0.05, Figure 5B). The contents of Cr and BUN, which are negatively correlated with the function of the kidney, were favorable in these results at either 8 or 12 w (Figures 5C, D).

A histological analysis was conducted at 8 and 12 w post-operation. The results of the HE and Masson staining showed that the PP46-gel scaffolds disappeared at 12 w while PP46 was degrading and some debris were still exhibited (Figure 5E). The collagen formed (blue in Figure 5E, Masson) and replaced the implanted scaffold. In contrast, the ureter substituted by PP46 showed that serious fibrosis with less urothelialization occurred and the scar formed at the surgery site. This fibrosis might be caused by excessive collagen secretion due to the dysurothelialization at the initial stage and prolonged duration of inflammation (Liu et al., 2020). Unexpectedly, compared with subcutaneous implantation (Figure 4A), a more severe and durable inflammatory response occurred for both scaffold replacements at 8 w, which might be caused by exposure of the scaffold to urine.

Cytokeratin AE1/AE3 is used to evaluate the growth of urothelium (Liu et al., 2020). Through IHC analysis, many urothelial cells and a layer of urothelium were detected on the luminal surface in PP46-gel-treated animals, while a thinner urothelial layer grew in the PP46 group after 8 w post-operation (Figure 5E, arrows). The urothelium matured more at 12 w than at 8 w. It is obvious that the gelatin-grafted scaffold PP46-gel promoted urothelialization compared to the ungrafted PP scaffold, though the ureter of animals implanted by the autograft displayed the best urothelialization. PP46-gel significantly reduced the hydronephrosis and dilation of the ureter compared with PP46. This is beneficial to protecting the renal function.

In this part, the PP-gel scaffold is more beneficial to achieving early uroepithelization and helps to avoid the formation of excessive fibrosis and repair the defected ureter compared with the PP scaffold. To understand this result, we explored the biologic mechanisms of ureter regeneration.

### 3.4 Detection of the biologic mechanism

**FIGURE 6:**
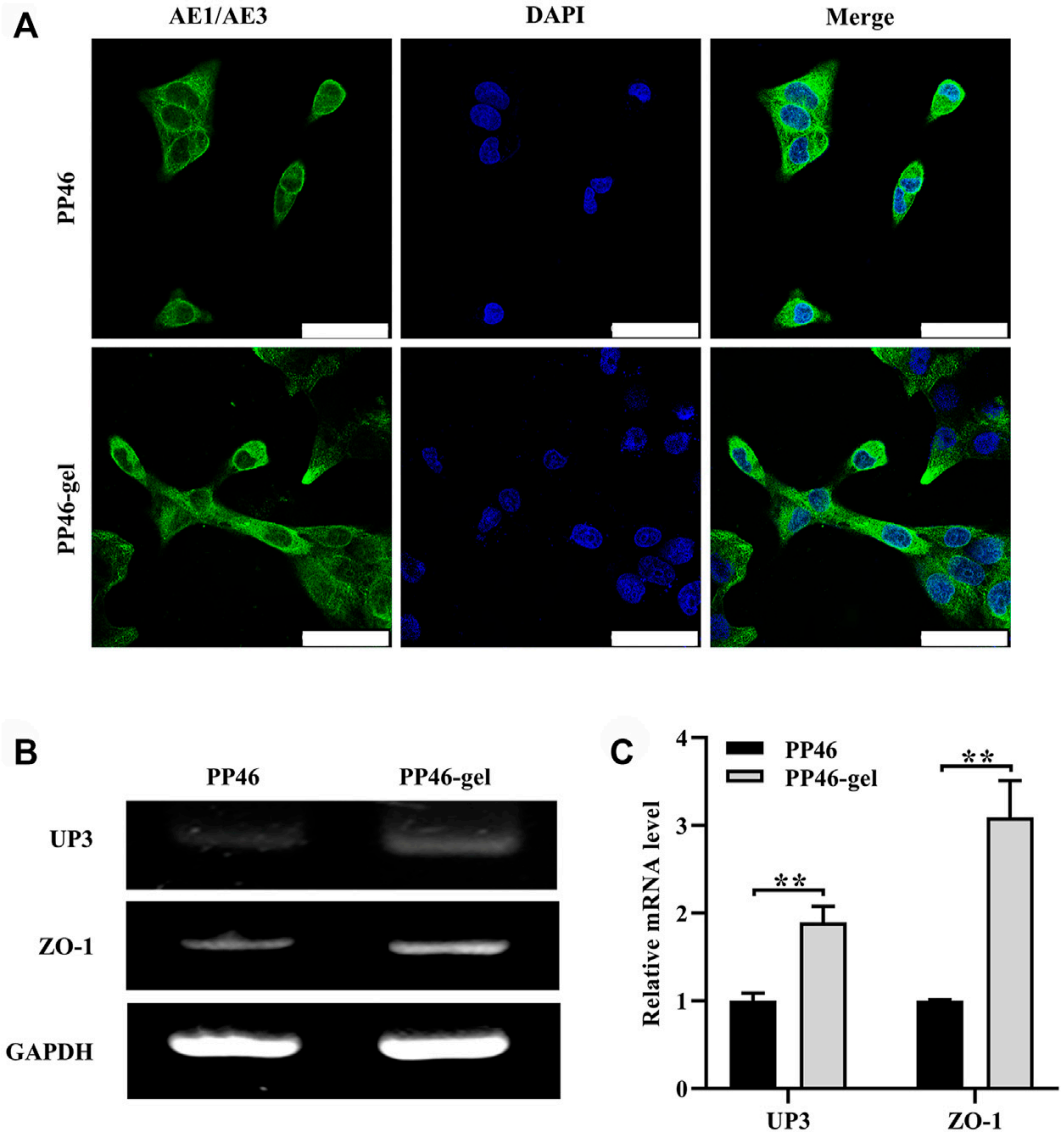
Phenotypic expression of HUCs on scaffolds PP46 and PP46-gel. **(A)** Epithelial phenotype with anti-cytokeratin AE1/AE3 as the primary antibody (green). The nuclei were stained with DAPI and displayed as blue. Cells were cultured for 7 days. Scale bar, 50 μm. **(B)** Analysis of UP3 and ZO-1 mRNA tested by RT-PCR. **(C)** Quantitative analysis of UP3 and ZO-1. Data are presented as mean ± SD (**p < 0.01).

Engineering the 3D ureter is a promising strategy for the treatment of stenosis-related diseases, especially if this 3D ureter can be epithelialized completely on the surface (Xie et al., 2021). In this work, we constructed a tubular asymmetric scaffold in which smaller micro-scale pores on the surface and larger pores in the bulk were fabricated using a mixture of biodegradable PLLA and PLCL as the matrix. Then, gelatin was grafted on the scaffold to improve its hydrophilicity and biocompatibility. The scaffold played a role in transporting urine with a similar biological function to the autografted ureter. To detect the biologic mechanism of how the scaffold works, the expressions of related proteins were investigated after the urothelial cells were seeded on the scaffolds. The results showed that more cells lived on PP46-gel than on PP46 after cells were cultured for 5 days. These cells maintained the urothelial cell phenotype (green fluorescence stained by IF with cytokeratin AE1/AE3 as the primary antibody) as shown in Figure 6A.

ZO-1 and UP3 are urothelial membrane protein representatives with a tight junction and AUM (Dalghi et al., 2020). Their expressions were analyzed using qRT-PCR technology at the mRNA level. The results showed that both of them were secreted more by cells on the PP46-gel than those on PP46 at day 7 (Figures 6B, C), which demonstrated that the gelatin-grafted scaffold had a pro-urothelium ability, including cell proliferation and expression of uroepithelial function-related genes.

**FIGURE 7:**
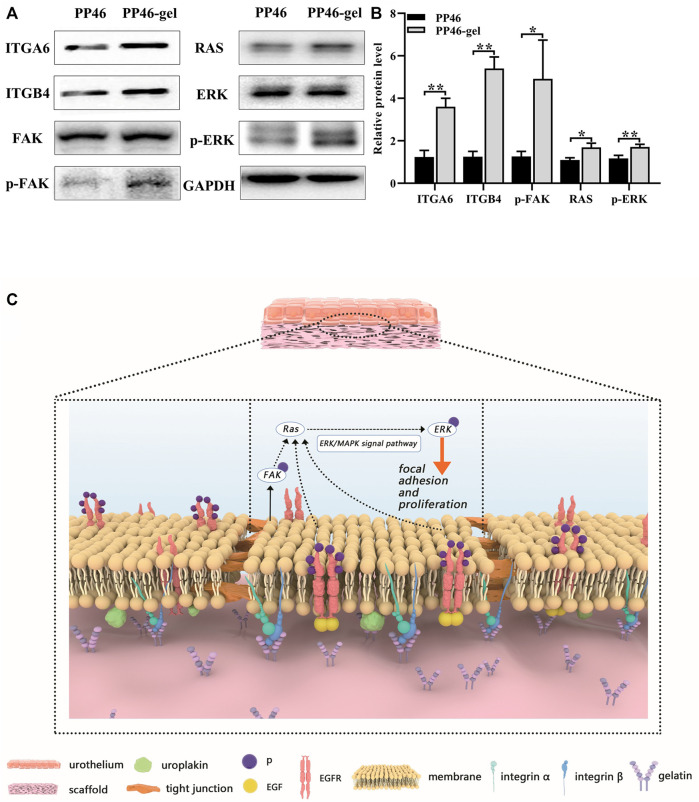
Studies of biological mechanisms. **(A)** Protein levels of ITGA6, ITGB4, FAK, p-FAK (Tyr 397), Ras, ERK, and p-ERK detected by Western blotting with GAPDH as the control. **(B)** Statistical analysis of ITGA6, ITGB4, p-FAK, Ras, and p-ERK. Cells were cultured for 7 days. **(C)** Schematic diagram regarding the molecular mechanism of the PP46-gel scaffold acting on the urothelial cells. Data are presented as mean ± SD (*p < 0.05; **p < 0.01).

In cell proliferation tests (Section 3.2), we detected that the gelatin-grafted scaffold promoted the proliferation of urothelial cells *via* mediating the G2/M stage of the cell cycle. Then, we speculated that the biological activity might be initiated by the interaction between the RGD sequence from gelatin and integrins on the cell membrane. Using qRT-PCR tests, we discovered the expressions of epithelial cell-specific integrins (integrin α6 and β4) and the downstream Ras genes (H-Ras, K-Ras and N-Ras). Integrin α6 (ITGA6), integrin β4 (ITGB4), K-Ras, and N-Ras genes were all activated, which indicated that urothelium promotion might be induced by the activation of the FAK/Ras related pathway (Supplementary Figure S5). Furthermore, the related proteins translated by these genes on this FAK/Ras signaling pathway were investigated by Western blot technology. Consistent results with qRT-PCR testing were also obtained (Figure 7A). The relative levels of proteins ITGA6, ITGB4, and Ras secreted by cells cultured for 7 days were higher on the gelatin-modified scaffold and PP46-gel than those on PP46 (Figures 7A, B). Moreover, the upgraded phosphorylation of proteins FAK and Erk (p-FAK and p-Erk in Figures 7A, B) suggest that the integrin/Erk signaling pathway was activated when urothelial cells were cultured on scaffolds, and much more on the PP46-gel than on PP46.

After speculating on the comprehensive effects of the PP46-gel scaffold on cell behaviors and the urothelialization *in vivo*, we propose the biologic mechanism about the biofunction improved by the gelatin-grafted scaffold as follows: integrin α6/β4 senses the RGD sequences from the gelatin-modified scaffold, and FAK is phosphorylated and the downstream MAPK/Erk pathway is activated. Additionally, the internal pores of the scaffold provide a good micro-environment to enroll more factors like EGF, which promotes cell infiltration and proliferation from the native urothelium. The urothelialization is thereby achieved, which enhances the tight junction among cells. Finally, both the tight junction among cells and the AUM on the cell membrane block harm from urine or other organisms on the deeper tissue like the submucosa. This molecular pathway is diagrammatically drawn in Figure 7C.

## 4 Conclusion

We successfully fabricated a tubular asymmetric scaffold with small micro-scale pores on the surface and larger pores in the bulk. Gelatin was grafted onto the scaffold using the method of diamine aminolysis and glutaraldehyde crosslinking. The resulting PP46-gel tubular scaffold is hydrophilic and biocompatible. A ureteral repair experiment in New Zealand rabbits showed that the gelatin-grafted scaffold greatly promoted urothelialization and ureteral regeneration, which protects ureteral and renal function. HUCs cultured on PP46-gel scaffolds exhibited normal cell morphology, which is possibly due to the RGD sequence of the grafted gelatin. Moreover, integrin α6/β4 on the cell membrane senses the RGD sequence and then phosphorylates FAK protein, which activates the downstream MAPK/Erk pathway in promoting cell adhesion and proliferation. Urothelialization and epithelium regeneration were finally achieved. In addition, more paracrine EGF in the PP46-gel group accelerates cells to start the growth life cycle. The consistency of these results confirmed that the PP46-gel scaffold is a promising candidate for the constitution of an engineered ureter and to repair long-segment ureteral defects.

## Data Availability

The original contributions presented in this study are included in the article/Supplementary Material, and further inquiries can be directed to the corresponding authors.
