# Supplementary material for: Gelatin-grafted tubular asymmetric scaffolds promote ureteral regeneration via activation of the integrin/Erk signaling pathway
Source: Front Bioeng Biotechnol. 2023 Jan 5;10:1092543. doi: 10.3389/fbioe.2022.1092543 (PMC9849368; doi:10.3389/fbioe.2022.1092543)
Supplement: Supplementary file 1 [file DataSheet1.docx]

Supplementary Material


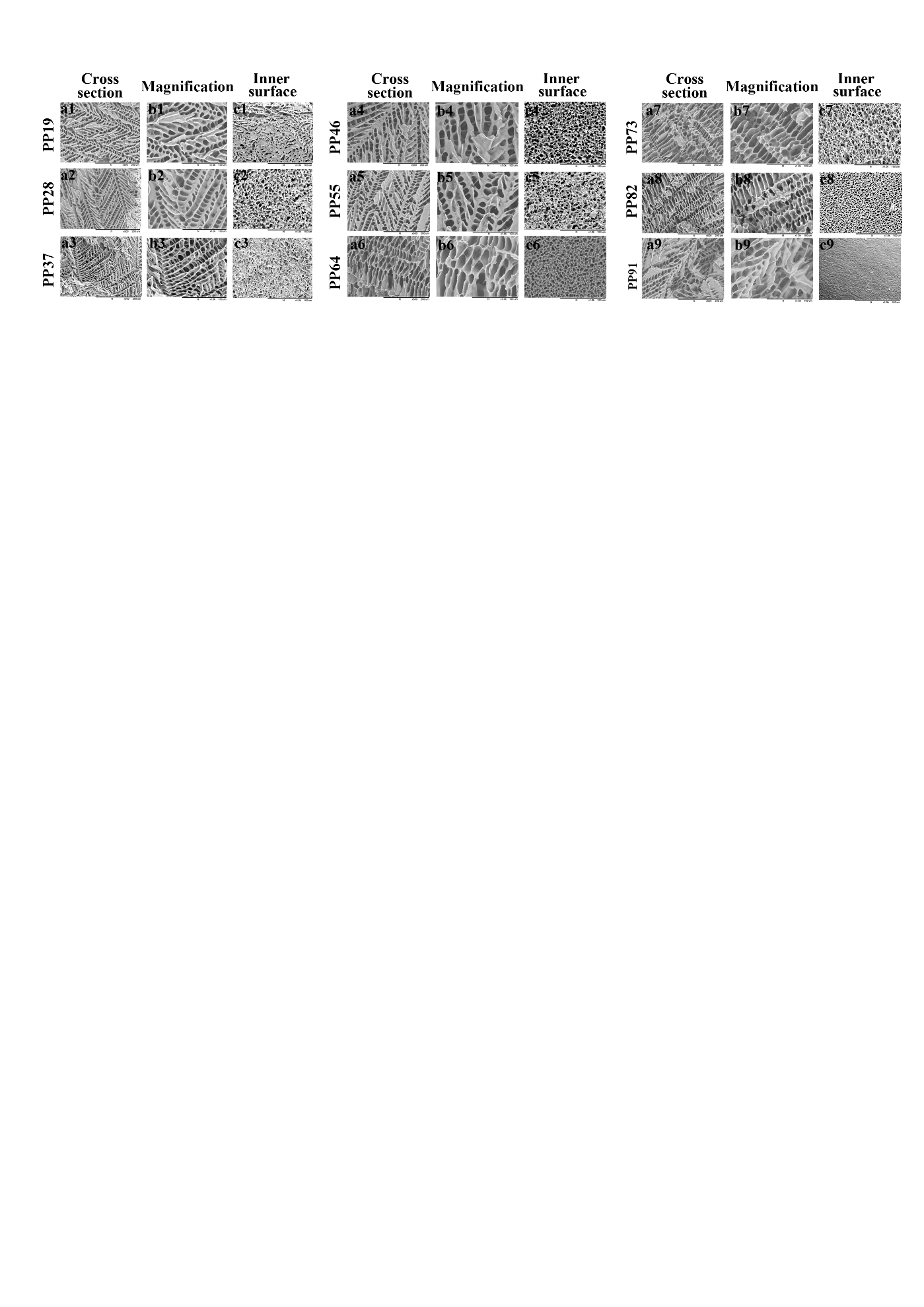


**Supplementary Figure S1** The morphology of scaffolds PP19-PP91, cross-section and inner surface, observed under SEM. (a1-a9) Cross sections. (b1-b9) Magnified from a1-a9, correspondingly. (c1-c9) The inner surfaces.

**
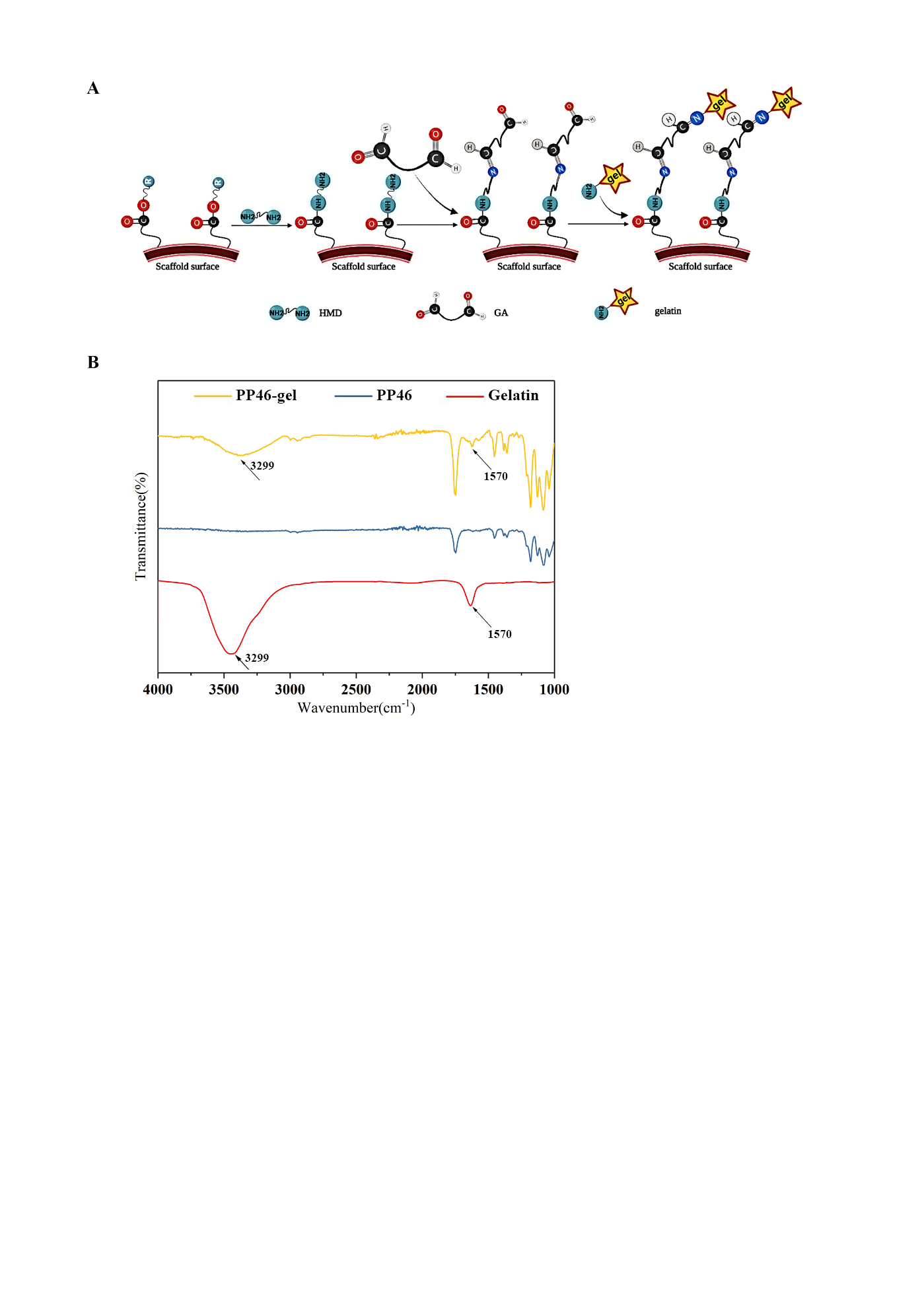
**

**Supplementary Figure S2** (A) Schematic reaction of gelatin grafting onto scaffold surface. (B) FTIR spectra of the ungrafted and grafted PLLA/PLCL scaffold as well as gelatin powder. The arrow denotes characteristic absorption peaks of gelatin.


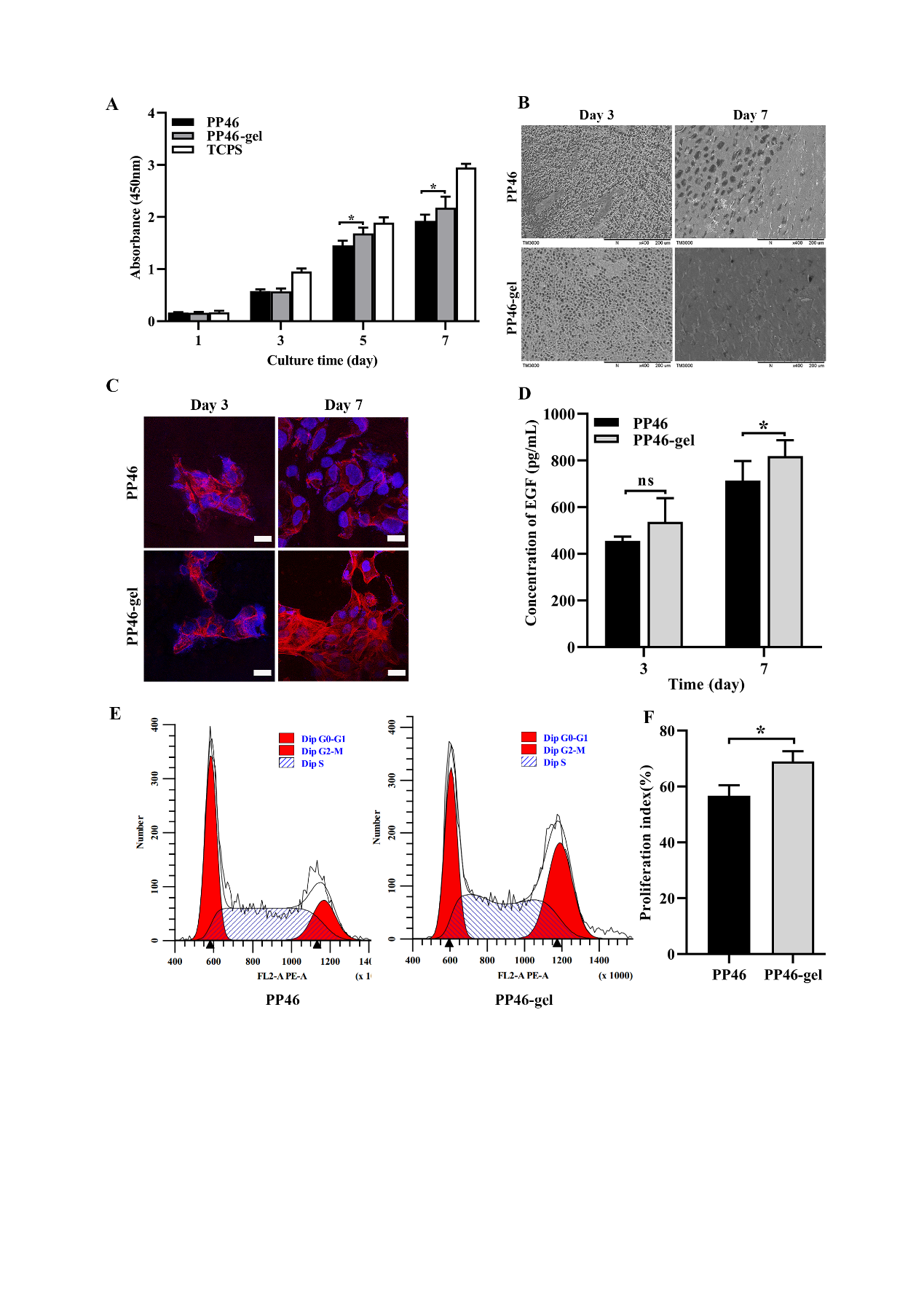


**Supplementary Figure S3** Absorbance at the wavelength of 450 nm, measured with CCK-8 assay. Cells were cultured for 7 days. **p*<0.05.


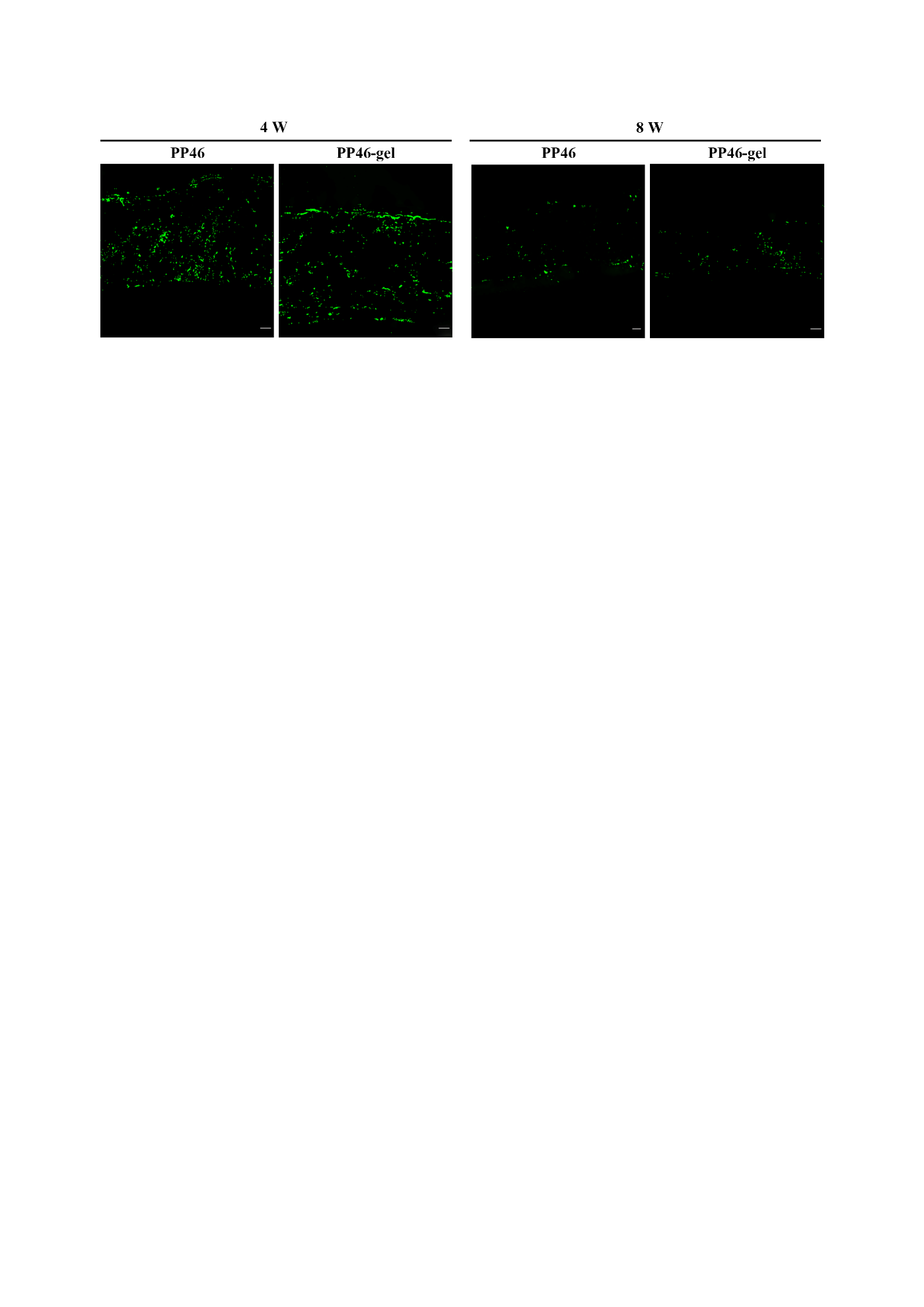


**Supplementary Figure S4** Immunofluorescent staining with anti-CD68 as the primary antibody, cross-sections. Scale bar, 500μm


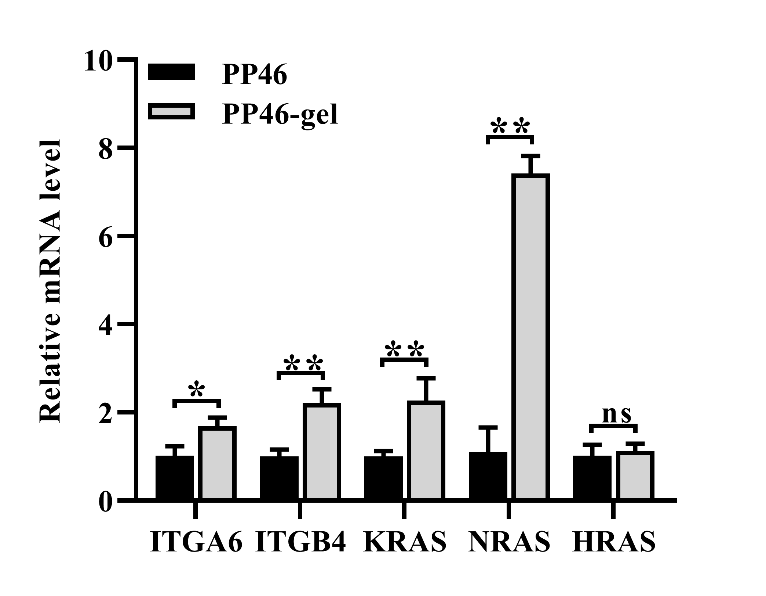


**Supplementary Figure S5** mRNA expression levels of ITGA6, ITGB4, K-Ras, N-Ras and H-Ras. ns: *p*>0.05, **p*<0.05; ***p<*0.01.

**Supplementary Table S1** Sequences for RT-PCR primers.

| **Primer** | **Sequences** |
| --- | --- |
| ZO-1 | F5'-CTGGTGAAATCCCGGAAAAATGA-3'  R5'-TTGCTGCCAAACTATCTTGTGA-3' |
| UP3 | F5'-CGTGGACATGGGGAGTTCTG -3'  R5'-TCACGGACGTGTAGGAAGACT-3' |
| integrin α6 | F5'-CAGTGGAGCCGTGGTTTTG-3'  R5'-CCACCGCCACATCATAGCC-3' |
| Integrin β4 | F5′-GCTTCACACCTATTTCCCTGTC-3′  R5′-GACCCAGTCCTCGTCTTCTG-3′ |
| K-Ras | F5'-ACAGAGAGTGGAGGATGCTTT-3'  R5'-TTTCACACAGCCAGGAGTCTT-3' |
| N-Ras | F5'-ATGACTGAGTACAAACTGGTGGT -3'  R5'-CATGTATTGGTCTCTCATGGCAC-3' |
| H-Ras | F5′- ATGACGGAATATAAGCTGGTGGT-3′  R5′-GGCACGTCTCCCCATCAATG-3′ |
| GAPDH | F5'-GGAGCGAGATCCCTCCAAAAT-3'  R5'-GGCTGTTGTCATACTTCTCATGG-3' |
